# Characterization of Ets-1 deficiency-induced depigmentation in a mouse model: insights into vitiligo pathogenesis

**DOI:** 10.1186/s42826-025-00260-8

**Published:** 2025-11-28

**Authors:** Wen-Yu Chang, Tzong-Shyuan Tai, Yu-Chun Lin, Po-Han Chen, Chih-Yang Chang, Yue-Chiu Su, Ying-Hsien Kao

**Affiliations:** 1 Department of Dermatology, E-Da Cancer Hospital, I-Shou University, Kaohsiung, 82445 Taiwan; 2 School of Medicine for International Students, College of Medicine, I-Shou University, Kaohsiung, 82445 Taiwan; 3 Advanced Immunology Laboratory, Chang Gung Memorial Hospital, Taoyuan, 33305 Taiwan; 4 Division of Reproductive Endocrinology and Infertility, Department of Obstetrics and Gynecology, E-Da Hospital, I-Shou University, Kaohsiung, 82445 Taiwan; 5 Department of Medical Research, E-Da Hospital, I-Shou University, Kaohsiung, 82445 Taiwan; 6 School of Medicine, College of Medicine, I-Shou University, Kaohsiung, 82151 Taiwan; 7 Department of Pathology, Kaohsiung Show Chwan Memorial Hospital, Kaohsiung, 80708 Taiwan

**Keywords:** Bioinformatics, Depigmentation, Melanogenesis, Next-generation RNA sequencing, Vitiligo

## Abstract

**Background:**

Vitiligo is a skin disorder characterized by the loss of melanocytes (MCs), leading to depigmentation. While the exact mechanisms are unclear, the transcription factor Ets-1, known for its role in regulating matrix metalloproteinase expression and MC migration, is suspected to play a part. Ets-1 gene-deficient mice exhibit a vitiligo-like phenotype with spontaneous skin depigmentation, suggesting a direct link between Ets-1 deficiency and MC dysfunction. This study aimed to characterize the molecular and histological features of Ets-1 gene knockout (KO) mice to understand the underlying mechanisms of this depigmentation.

**Results:**

Transcriptomic analysis of depigmented and normal skin from Ets-1 KO and wild-type mice revealed significant differentially expressed genes (DEGs). KEGG pathway enrichment analysis demonstrated alterations in metabolic and signal transduction pathways, notably the downregulation of melanogenesis-related genes. RT-qPCR and immunohistochemistry confirmed reduced tyrosinase expression at both transcript and protein levels in the depigmented skin of KO mice. Protein-protein interaction network analysis of the DEGs highlighted a central network involving keratin proteins and discrete interactions regulating melanogenesis, stress response, and cellular signaling pathways.

**Conclusions:**

These findings demonstrate that Ets-1 deficiency in mice leads to significant molecular and histological changes consistent with MC dysfunction and depigmentation. The observed downregulation of melanogenesis-related genes and alterations in key signaling pathways provide valuable insights into the molecular basis of Ets-1’s role in MC maintenance and suggest potential therapeutic targets for skin pigmentation disorders, including vitiligo.

**Supplementary Information:**

The online version contains supplementary material available at 10.1186/s42826-025-00260-8.

## Background

Vitiligo vulgaris is a common depigmentation disorder caused by the destruction of functional melanocytes (MCs) in the affected skins of patients. To date, several prevailing pathogenetic hypotheses have been proposed for human vitiligo, primarily involving the interplay among intrinsic MC defects, environmental factors, and autoimmune mechanisms [1, 2]. Given the limited efficacy of conventional therapies—such as psoralens plus ultraviolet light irradiation, as well as systemic or topical steroids [3, 4]—there is an urgent need to develop more effective therapeutic approaches for vitiligo treatment. Numerous animal models exhibiting spontaneous or induced patchy skin depigmentation have been reported in previous studies [1]. These preclinical models may serve as powerful tools for investigating the pathogenesis of vitiligo and developing novel therapeutic strategies.

Ets-1, a prototype of the ETS family of transcription factors, structurally features a DNA-binding domain at its C-terminal [5]. The transcription of Ets-1-regulated genes is dependent on the phosphorylation of a conserved threonine residue, a process mediated by extracellular signal-regulated kinase (ERK) [6]. Additionally, Ets-1 interacts with numerous nuclear co-activators to drive gene-specific responses that regulate distinct biological processes, including lymphoid organ development [5, 7]. Genetic aberrations in Ets-1 have been implicated in the pathogenesis of autoimmune diseases [8], as well as in the progression of leukemia and solid tumors [9, 10]. It has long been established that, in response to signals conveyed by ERK and c-Jun N-terminal kinase, Ets-1 binds to the promoters of matrix metalloproteinase (MMP) genes and activates their transcription in tumor cells [11]. Moreover, the association between elevated Ets-1 expression and melanoma malignancy underscores it critical role in promoting melanoma cell migration [12], further suggesting its regulatory function in normal MCs and/or melanoblasts.

**Fig. 1:**
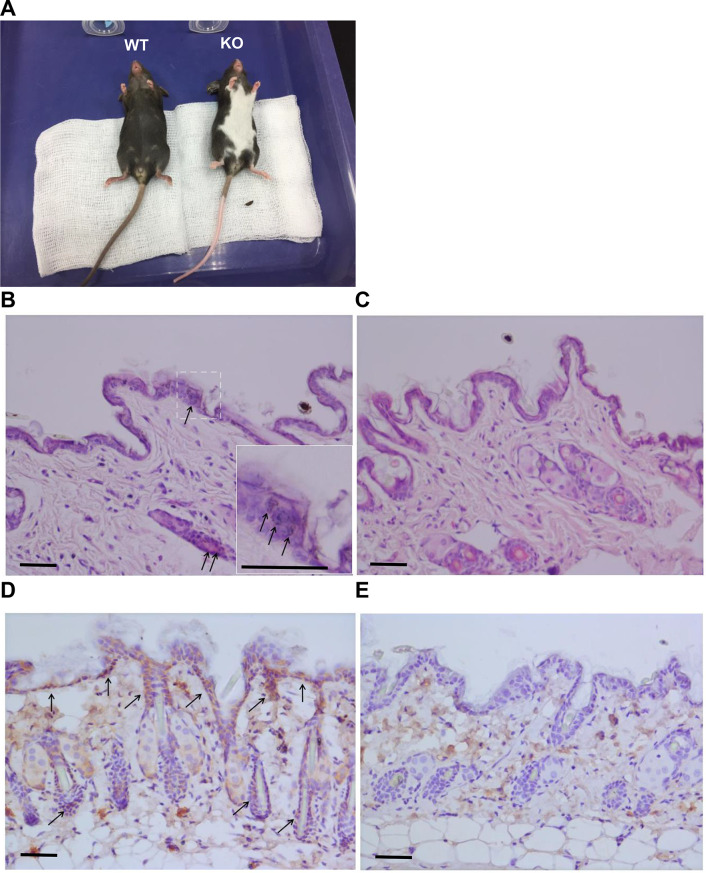
Phenotypic and histological characterization of Ets-1 wild-type (WT) and knockout (KO) mice. (**A**) Representative photographs of WT (left) and KO (right) mice at 10-week old, showing the depigmentation phenotype in KO mice. (**B**, **C**) Hematoxylin and eosin staining of skin sections from Ets-1 WT (**B**) and KO (**C**) mice. Arrows and the zoomed-in inset indicate melanosome granules within keratinocytes of the epidermis and outer root sheath of hair follicles. (**D**, **E**) Immunohistochemistry staining for Ets-1 protein in skin sections of WT (**D**) and KO (**E**) mice. Arrows indicate Ets-1 expression in keratinocytes of the epidermis and outer root sheath in WT mice, which is absent in KO mice. Note that the pseudo-positive signal in the dermis is likely due to endogenous mouse IgG deposition in skin tissues. Bars, 100 μm

In the context of vitiligo pathogenesis, Ets-1 downregulation has been previously reported in the skins of vitiligo patients. Despite neither transcription nor translation of Ets-1 was detectable in the MCs isolated from vitiligo patients, the Ets-1 downregulation is proposed to underlie the decreased expression and activity of MMPs [13] and the reduced expression of cell adhesion molecules [14]. In the immunoregulatory aspect, dysregulation of interleukin-17 (IL-17) and subsequent immune responses are recognized as characteristic features in both human vitiligo and mouse models of the disease [15, 16]. In this regard, Ets-1 gene knockout (KO) mice exhibit abnormally high levels of IL-17 transcripts in their lungs and circulation, along with increased mucus production by airway epithelial cells in an IL-17-dependent manner [17]. Notably, all Ets-1 KO mice spontaneously develop a distinct area of white hair on their bellies and depigmentation of distal limbs, including the feet and tails (Fig. 1A). Based on these observations, we proposed that the Ets-1 KO mice may serve as a suitable model for studying the pathomechanisms of vitiligo and developing therapeutic strategies. Therefore, this study aimed to compare the transcriptome of depigmented skins from KO moue with that of pigmented skin from the same mouse, as well as skin from wild-type (WT) mouse. Additionally, we validated mRNA alterations in melanogenesis-related genes and examined the histological characteristics of the depigmented mouse skin.

## Methods

### Animals

Adult male WT C57BL/6 mice, weighing 25–30 g, were purchased from BioLASCO (Taipei, Taiwan). Ets-1 KO mice, previously established with an N5 C57BL/6 genetic background [18], were bred and housed in the Animal Center of I-Shou University. Skin samples from male mice were collected under deep anesthesia, followed by euthanasia, in accordance with a protocol approved and monitored by the Institutional Animal Care and Use Committee (IACUC) of E-Da Hospital (Approval nos. IACUC-EDCH-107002 and 108004).

### Transcriptome analysis by next-generation sequencing (NGS)

Total RNA of mouse skin tissues was isolated using Trizol Reagent (Invitrogen, Carlsbad, CA, USA) following the manufacturer’s instructions. The purified RNA was then sent to Welgene Biotech (Taipei, Taiwan) for custom RNA-seq services. RNA quality was assessed using a Bioanalyzer 2100 (Agilent Technologies, Palo Alto, CA, USA) with an RNA 6000 LabChip kit (Agilent Technologies) and quantified by measuring the optical density at 260 nm using a spectrophotometer (ND-1000, Nanodrop Technology, USA). RNA libraries were constructed using the SureSelect Strand-Specific RNA Library Preparation Kit (Agilent), followed by size selection with AMPure XP beads (Beckman Coulter, USA). Raw sequences were generated using a NGS platform (HiSeq 2500, Illumina, San Diego, CA, USA) based on sequencing-by-synthesis (SBS) technology, with 300-cycle paired-end reads at a depth of 20 million reads per sample. Paired-end sequencing data (FASTQ reads) were produced using base-calling conversion software (bcl2fastq v2.20, Illumina). Adaptor clipping and sequence quality trimming were performed using a Trimmomatic software (v0.36, Illumina). The sequence reads were aligned to annotated transcripts in the Genome Reference Consortium Mouse Build 38 patch release 5 (GRCm38.p5) using HISAT2 program. The unprocessed RNA-seq read mapping files have been deposited in the ArrayExpress database (Accession no. E-MTAB-12835).

### Enrichment analysis and KEGG pathways

Analysis of differentially expressed genes (DEGs) was conducted using Cuffdiff (Cufflinks 2.2.1) with genome bias detection and correction through in-house programs developed by Welgene Biotech. The expression levels of DEGs were quantified as fragments per kilobase of transcript per million mapped reads (FPKM) and normalized by calculating Log2 ratios between groups. P values were determined using a hypergeometric distribution to assess the probability of random sampling and significance was adjusted for false discovery rate (FDR). The DEGs identified from group comparisons were subjected to functional enrichment analysis using the ClusterProfiler 3.6 package, with adjusted p-values (q-values) calculated using the Benjamini-Hochberg procedure.

### Reverse transcription and quantitative polymerase chain reaction (RT-qPCR)

The significant DEGs in the melanogenesis pathway, identified through transcriptomic RNA-seq analysis, were validated using the RT-qPCR method. Briefly, total RNA extracted from mouse skins was reverse-transcribed into cDNA using an iScript cDNA synthesis kit (Bio-Rad, Hercules, CA, USA). The cDNA was then amplified and quantified using a SYBR Green Master Mix kit (ABI, Foster City, CA, USA) on a real-time PCR machine (Eco qPCR, Illumina, San Diego, CA, USA). The primer sequences used for qPCR are listed in Supplementary Table S1. Relative expression levels were calculated by Ct method and normalized to β-actin reference gene, which served as an internal loading control.

### Histological examination and immunohistochemistry (IHC)

Formalin-fixed and paraffin-embedded mouse skin tissues were sectioned and used for hematoxylin and eosin (H&E) and IHC staining, as previously described [19]. Briefly, tissue sections were deparaffinized, rehydrated, and subjected to antigen retrieval by microwave treatment in citrate buffer (pH 6.0). Endogenous mouse IgG was blocked using 50 µg/mL goat anti-mouse IgG (Cat. No. 115-007-003, Jackson Laboratories, West Grove, PA, USA) in background reduction diluent (Cat. No. S3022, Dako, Glostrup, Denmark). Sections were then incubated overnight at 4 °C with the following primary antibodies: rabbit monoclonal anti-Ets-1 (Cat. No. 14069, Cell Signaling Technology, Danvers, MA, USA) and mouse monoclonal anti-tyrosinase (Tyr) (Cat. No. sc-20035, Santa Cruz Biotechnology, Santa Cruz, CA, USA). Antigen localization was visualized using a HRP-conjugated EnVision detection system (Cat. No. K5007, DAKO), followed by hematoxylin counterstaining. For negative controls, normal skin sections from WT mice were incubated with isotype-matched control IgG at equimolar concentrations.

### Protein-protein interaction (PPI) network construction

To explore the potential roles of DEGs identified in the depigmented skin of KO mice and the normal skins of both KO and WT mice, PPI networks were constructed and visualized using Cytoscape (http://www.cytoscape.org/), based on data from the STRING database. Clustering analysis was performed using the MCODE plugin to identify hub genes and construct functional modules. The analysis was conducted with the following settings: MCODE score > 2, degree cutoff = 2, node score cutoff = 0.2, k-core = 2, and maximun depth = 100. This approach allows for the identification of key regulatory networks and functional modules associated with the observed phenotypic changes in Ets-1 KO mice.

### Statistics

Data are presented as the mean ± standard error of the mean (SEM) and are representative of at least three individual animal skin specimens. Statistical analysis was performed using GraphPad Prism 7.0 (GraphPad Software, La Jolla, CA, USA). Differences between groups were assessed using a 2-tailed Student’s t-test or ANOVA, followed by Dunnett’s post hoc test for multiple comparisons. A p-value of less than 0.05 was considered statistically significant and is indicated by asterisks in the figures.

## Results

### Skin depigmentation in Ets-1 KO mice

As previous studies’ findings suggest that Ets-1 downregulation in vitiligo-affected skin may contribute to the depigmented phenotype observed in vitiligo [13, 14], we hypothesize the Ets-1 gene-deficient mice could serve as a suitable model for studying vitiligo pathogenesis and developing therapeutics for skin depigmentation disorders. All adult Ets-1 KO mice exhibited characteristics consistent with vitiligo, including a distinct area of white hair on the belly and depigmentation of distal limbs, such as feet and tails (Fig. 1A).

### Histological observations

To investigate histological differences between normal and depigmented skins, we performed H&E staining followed by microscopic observation. In the skin of Ets-1 KO mice, melanosome granules were significantly reduced in keratinocytes within both the epidermis and the outer root sheath of hair follicles (Fig. 1B and C). IHC staining confirmed widespread expression of Ets-1 protein in keratinocytes of the epidermis and hair follicle outer root sheath in WT mice, while Ets-1 was absent in the corresponding cells of Ets-1 KO mice (Fig. 1D and E).

### Comparative transcriptomic analysis by NGS RNA-sequencing

To compare the transcriptomic profiles between the two genotypes, total RNAs were collected from the normal skins (with black hair coats) of both WT and KO mice, as well as from the depigmented skins of Ets-1 KO mice, and subjected to RNA-seq transcriptomic analysis. The transcriptome profiles of the three mouse skin tissues were compared, and DEGs were analyzed. Fragment signals mapped to immunoglobulin variable and joint genes, T cell receptor variable genes, predicted genes, pseudogenes, and unspecified RIKEN cDNA fragments were excluded from the analysis. When compared to the transcriptome of WT mouse skin, 223 DEGs were identified in the normal skins of Ets-1 KO mice, including 139 upregulated genes and 84 downregulated genes (Table 1 lists the top 10 upregulated and downregulated DEGs). In contrast, the transcriptome of depigmented skins of Ets-1 KO mice, when compared to normal mouse skins, revealed only 52 DEGs, consisting of 15 upregulated genes and 37 downregulated genes (Table 2 lists the top 10 upregulated and downregulated DEGs). Additionally, transcriptome comparison between depigmented and pigmented skins of KO mice identified a total of 148 DEGs, including 42 upregulated genes and 106 downregulated genes (Table 3 lists the top 10 upregulated and downregulated DEGs).

**Table 1:** Top 10 upregulated and downregulated protein-coding genes in the pigmented skins of Ets-1 KO mice compared to WT mouse skins

Gene symbol	Gene ID	Gene name	Log2 ratios*	*P*-value
Upregulated genes				
* Crisp1*	11,571	Cysteine-rich secretory protein 1	20.267566246	0.00005
* Srd5a2*	94,224	Steroid 5 alpha-reductase 2	17.169347635	0.00005
* Krtap5-1*	50,774	Keratin associated protein 5 − 1	16.877080389	0.00005
* Crisp3*	11,572	Cysteine-rich secretory protein 3	16.845735053	0.00015
* Atoh7*	53,404	Atonal bHLH transcription factor 7	15.262384853	0.00015
* Slc30a3*	22,784	Solute carrier family 30, member 3	15.130896290	0.00015
* Fgf3*	14,174	Fibroblast growth factor 3	15.061134886	0.00045
* Krtap2-4*	71,453	Keratin associated protein 2–4	6.821849303	0.00055
* Krtap16-1*	100,504,183	Keratin associated protein 16 − 1	6.396767116	0.00005
* Tgm6*	241,636	Transglutaminase 6	6.381036326	0.00045
Downregulated genes				
* Klra3*	16,634	Killer cell lectin-like receptor, subfamily A, member 3	-17.362312005	0.00005
* Klri1*	503,550	Killer cell lectin-like receptor family I member 1	-16.959028400	0.0001
* Klra4*	93,970	Killer cell lectin-like receptor, subfamily A, member 4	-16.729647294	0.0001
* Fgl1*	234,199	Fibrinogen-like protein 1	-16.643658932	0.0003
* Ncr1*	17,086	Natural cytotoxicity triggering receptor 1	-16.578783963	0.00005
* Klra7*	16,638	Killer cell lectin-like receptor, subfamily A, member 7	-16.567731824	0.00005
* Klri2*	320,407	Killer cell lectin-like receptor family I member 2	-16.423022323	0.00005
* Otx1*	18,423	Orthodenticle homeobox 1	-15.902163104	0.00005
* Defb8*	244,334	Defensin beta 8	-7.685888094	0.00025
* Serpinb6d*	238,568	Serine peptidase inhibitor, clade B, member 6d	-5.834049692	0.00005

*Fold changes of differentially expressed genes are calculated and presented as Log2 ratios

**Table 2:** Top 10 upregulated and downregulated protein-coding genes in depigmented skins of Ets-1 KO mice compared to WT normal skins

Gene symbol	Gene ID	Gene name	Log2 ratios *	*P*-value
Upregulated genes				
* Fam183b*	75,429	Family with sequence similarity 183, member B	16.796255124	0.00025
* Atp6v1g3*	338,375	ATPase, H + transporting, lysosomal V1 subunit G3	16.303777181	0.00005
* Col6a5*	665,033	Collagen, type VI, alpha 5	3.8041388417	0.00005
* Ctse*	13,034	Cathepsin E	3.5197245922	0.00040
* Fos*	14,281	FBJ osteosarcoma oncogene	2.5826466791	0.00005
* Spon1*	233,744	Spondin 1, (f-spondin) extracellular matrix protein	2.4333697063	0.00005
* Egr1*	13,653	Early growth response 1	2.2376373197	0.00005
* Mrgprg*	381,974	MAS-related GPR, member G	1.9581840161	0.00005
* Junb*	16,477	Jun B proto-oncogene	1.9231498776	0.00005
* Dusp1*	19,252	Dual specificity phosphatase 1	1.8467506092	0.00005
Downregulated genes				
* Krtap16-3*	71,369	Keratin associated protein 16 − 3	-19.556178588	0.00005
* Krtap19-3*	77,918	Keratin associated protein 19 − 3	-18.507860438	0.00005
* Krtap4-7*	76,444	Keratin associated protein 4–7	-18.219154337	0.00005
* Krtap1-4*	629,873	Keratin associated protein 1–4	-18.149166583	0.00005
* Trat1*	77,647	T cell receptor associated transmembrane adaptor 1	-18.115450123	0.00005
* Klra3*	16,634	Killer cell lectin-like receptor, subfamily A, member 3	-17.362312005	0.00005
* Krtap4-8*	665,992	Keratin associated protein 4–8	-17.084227074	0.00005
* Krtap4-9*	665,998	Keratin associated protein 4–9	-17.073357085	0.0001
* Klri1*	503,550	Killer cell lectin-like receptor family I member 1	-16.959028400	0.0001
* Klra4*	93,970	Killer cell lectin-like receptor, subfamily A, member 4	-16.729647294	0.0001

*Fold changes of differentially expressed genes are calculated and presented as Log2 ratios

**Table 3:** Top 10 upregulated and downregulated protein-coding genes in depigmented skins of Ets-1 KO mice compared to paralesional pigmented skins

Gene Symbol	Gene ID	Gene name	Log2 ratios *	*P*-value
Upregulated genes				
* Otx1*	18,423	Orthodenticle homeobox 1	15.703089773	0.00005
* Defb8*	244,334	Defensin beta 8	6.0619398415	0.00025
* Serpinb6d*	238,568	Serine peptidase inhibitor, clade B, member 6d	5.1456323981	0.00005
* Sln*	66,402	Sarcolipin	3.6257767032	0.00005
* Cyp2b23*	243,881	Cytochrome P450, family 2, subfamily b, polypeptide 23	3.4418384680	0.00005
* Ceacam3*	384,557	Carcinoembryonic antigen-related cell adhesion molecule 3	2.8584292376	0.00045
* Sbk3*	381,835	SH3 domain binding kinase family, member 3	2.8031581896	0.00005
* Serpinb6c*	97,848	Serine peptidase inhibitor, clade B, member 6c	2.4770399675	0.00005
* Rnase2b*	54,159	Ribonuclease, RNase A family, 2B	2.4203273806	0.00005
* Cyp2j12*	242,546	Cytochrome P450, family 2, subfamily j, polypeptide 12	2.2909894575	0.00005
Downregulated genes				
* Krtap19-3*	77,918	Keratin associated protein 19 − 3	-24.449833020	0.00005
* Krtap4-7*	76,444	Keratin associated protein 4–7	-24.259413989	0.00005
* Krtap16-3*	71,369	Keratin associated protein 16 − 3	-24.165249595	0.00005
* Krtap1-4*	629,873	Keratin associated protein 1–4	-23.678386513	0.00005
* Krtap4-9*	665,998	Keratin associated protein 4–9	-23.031128047	0.00005
* Krtap19-4*	170,654	Keratin associated protein 19 − 4;	-23.030973192	0.00005
* Krtap4-8*	665,992	Keratin associated protein 4–8	-23.017202272	0.00005
* Krtap19-1*	170,657	Keratin associated protein 19 − 1	-22.210475157	0.00005
* Krtap4-13*	69,464	Keratin associated protein 4–13	-22.004919250	0.00005
* Krtap28-10*	70,831	Keratin associated protein 28 − 10	-21.465635153	0.00005

*Fold changes of differentially expressed genes are calculated and presented as Log2 ratios

**Fig. 2:**
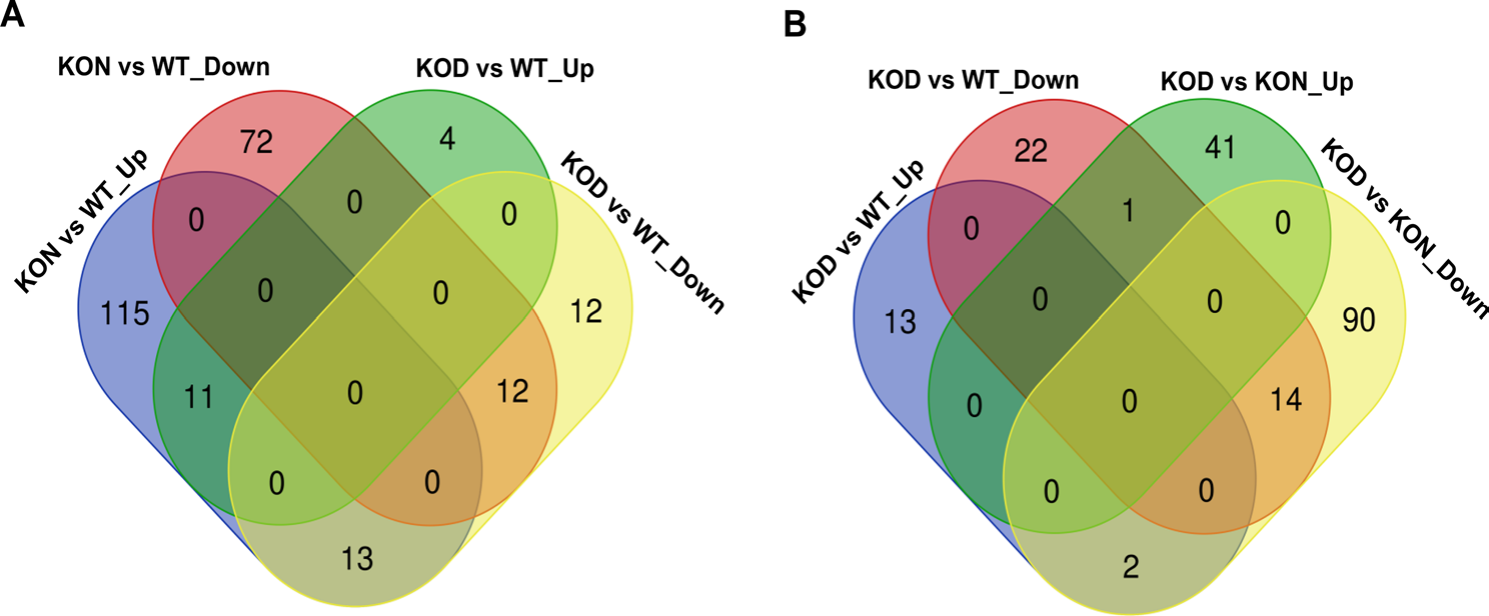
Venn diagrams analysis of differentially expressed genes (DEGs) identified by comparing between Ets-1 knockout (KO) and wild-type (WT) mice. (**A**) Overlap of DEGs in depigmented (KOD) and normal (KON) skins from KO mice compared to WT controls. (**B**) Overlap of DEGs in KOD skin compared to either KON or WT skins. The analysis was performed using RNA-seq data, and DEGs were identified with a threshold of |log2 fold change| >1 and adjusted p-vale < 0.05. Numbers within sections indicate the number of shared DEGs. “Up” and “Down” indicated upregulated and downregulated DEGs, respectively

To identify distinct and overlapping DEG patterns, a Venn diagram analysis was conducted to compare the DEG profiles in the transcriptomes of depigmented and normal skin tissues from KO and WT normal skin tissues. Compared to WT transcriptome, both normal and depigmented skin from KO mice shared 11 upregulated and 12 downregulated DEGs (Fig. 2A). Notably, four unique upregulated genes—*Fam183b*, *Cd209e*, *Mrgprg*, and *Atp6v1g3*—were identified exclusively in the depigmented skin, with no significant changes observed in KO normal skin, suggesting their potential role in driving depigmentation process. Conversely, comparison of the depigmented skin transcriptome with both WT and KO normal skin types revealed 14 shared downregulated DEGs between the two normal skin (Fig. 2B). Among these, 11 genes belonged to the keratin and keratin-associated protein (Krtap) families, implicating their involvement in depigmentation. Intriguingly, the *Defb8* gene exhibited contrasting regulation: it was downregulated compared to WT normal skin but significantly upregulated compared to KO normal skin. These findings underscore the functional complexity of DEGs in the context of depigmentation and skin homeostasis, highlighting key candidates for further mechanistic exploration.

### KEGG pathway enrichment analysis

KEGG pathway enrichment analysis was subsequently performed using the DEGs identified from comparisons between Ets-1 KO and WT mouse skins. The enrichment analysis of DEGs between depigmented and pigmented skins of Ets-1 KO mice revealed 11 significantly altered metabolic and signal transduction pathways (Q-values < 0.05; Table 4). Notably, melanogenesis pathway was also identified (Q value = 0.01699), supporting the involvement of impaired melanogenesis function in the depigmented skin of KO mice. Downregulated genes in the melanogenesis pathway included *Tyr*, tyrosinase-related protein 1 (*Tyrp1*), lymphoid enhancer binding factor 1 (*Lef1*), dopachrome tautomerase (*Dct*), frizzled class receptor 1 (*Fzd1*), endothelin receptor type B (*Ednrb*), and SRY (sex determining region Y)-box 9 (*Sox9*) (Table 5). Although the transcription of the microphthalmia-associated transcription factor (*Mitf*) gene was only slightly suppressed (*P* = 0.0518), these findings strongly suggests that Ets-1 gene deficiency significantly impacts the activation of melanogenesis-related genes (the visualized pathway is shown in Supplementary Fig. S1). Additionally, enrichment analysis between the normal skins of KO and WT mice identified 17 significantly altered metabolic and signal transduction pathways (Q-values < 0.05; Supplementary Table S2), with the melanogenesis pathway approaching significance (Q = 0.0557). Furthermore, the comparison between depigmented skin of KO mice and normal skin of WT mice revealed 16 altered pathways (Q-values < 0.05; Supplementary Table S3).

**Table 4:** KEGG pathway enrichment analysis on the differentially expressed genes (DEGs) in depigmented skins of Ets-1 KO mice compared with paralesional pigmented skins

Pathway	DEG no. (% of total 191 DEGs)	Genes in pathway (% total 8275 annotated genes)	*P*-value	Q-value *	Pathway ID
Estrogen signaling pathway	18 (9.42%)	133 (1.61%)	0.0000000012	0.0000002678	mmu04915
Retinol metabolism	11 (5.76%)	91 (1.10%)	0.0000070305	0.0007770514	mmu00830
Arachidonic acid metabolism	10 (5.24%)	89 (1.08%)	0.0000354254	0.0024118124	mmu00590
Ovarian steroidogenesis	8 (4.19%)	57 (0.69%)	0.0000436423	0. 0024118124	mmu04913
Fatty acid elongation	6 (3.14%)	32 (0.39%)	0.0000768056	0.0033956148	mmu00062
Melanogenesis	9 (4.71%)	100 (1.21%)	0.0004793549	0.0169944909	mmu04916
Mineral absorption	6 (3.14%)	45 (0.54%)	0.0005381589	0.0169944909	mmu04978
Steroid biosynthesis	4 (2.09%)	19 (0.23%)	0.0008122681	0.0224442497	mmu00100
Breast cancer	10 (5.24%)	147 (1.78%)	0.0020854820	0.0440576998	mmu05224
Tyrosine metabolism	5 (2.62%)	40 (0.48%)	0.0021226818	0.0440576998	mmu00350
Wnt signaling pathway	10 (5.24%)	148 (1.79%)	0.0021923951	0.0440576998	mmu04310

* Adjusted P-value depicting significant enrichment (Q-value < 0.05) of the gene sets in the pathways

**Table 5:** Downregulated genes in melanogenesis pathway (depigmented vs. pigmented skins of Ets-1 KO mice)

Gene symbol	Gene ID	Gene name	Log2 ratios *	*P*-value	KEGG Orthology
*Tyr*	22,173	Tyrosinase	-16.37359248	0.00005	K00505
*Tyrp1*	22,178	Tyrosinase-related protein 1	-7.309759118	0.04115	K00506
*Lef1*	16,842	Lymphoid enhancer binding factor 1	-5.289018911	0.0035	K04492
*Dct*	13,190	Dopachrome tautomerase	-5.119460116	0.0042	K01827
*Fzd1*	14,362	Frizzled class receptor 1	-1.644033562	0.00005	K02432
*Ednrb*	13,618	Endothelin receptor type B	-1.330469365	0.0012	K04198
*Sox9*	20,682	SRY (sex determining region Y)-box 9	-1.251634413	0.0093	K18435
*Mitf*	17,342	Microphthalmia-associated transcription factor	-0.760534770	0.0518	K09455

*Fold changes of differentially expressed genes are calculated and presented as Log2 ratios

### Validation of melanogenesis genes

**Fig. 3:**
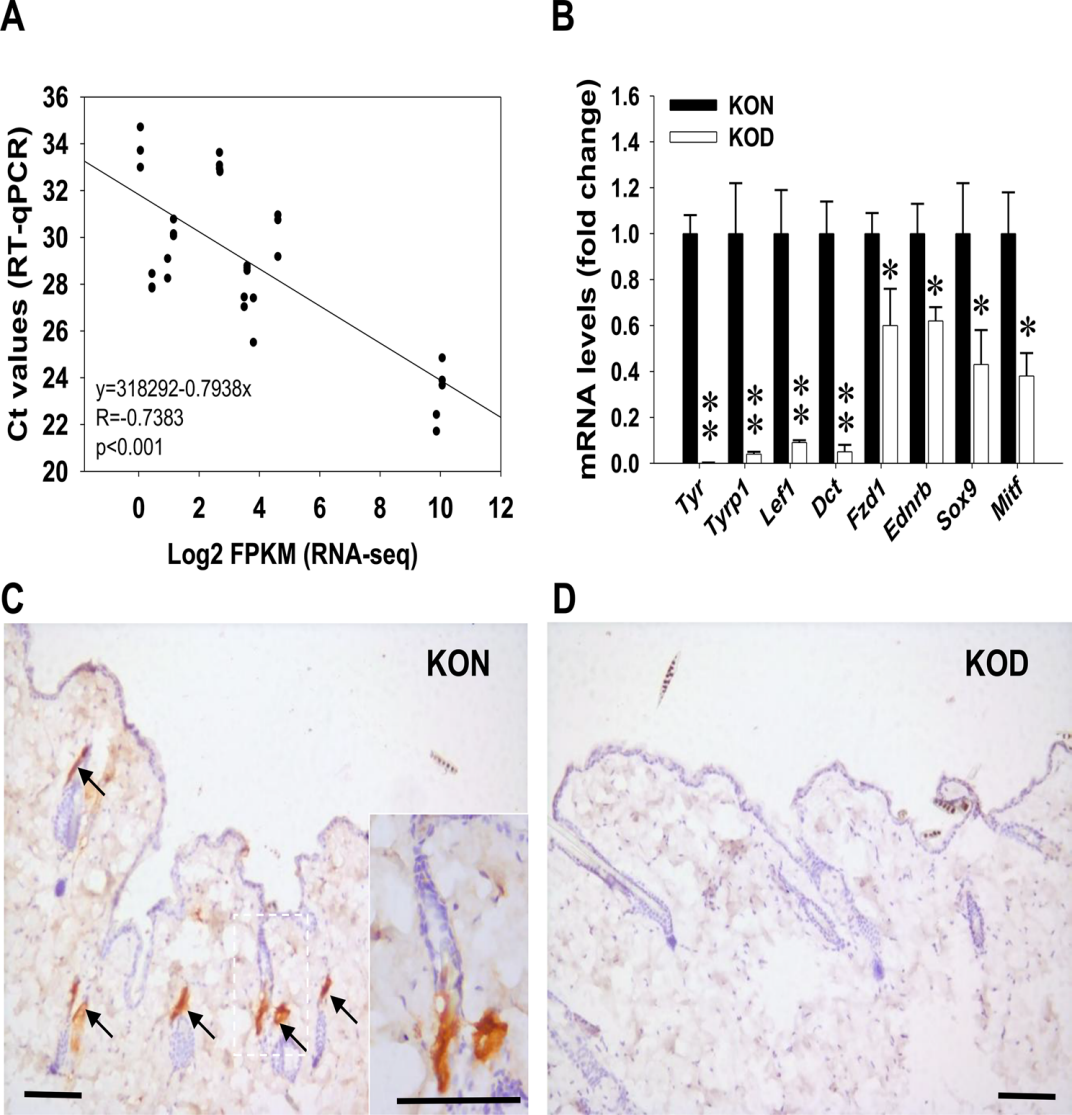
Reduced expression of melanogenesis related genes and tyrosinase protein in Ets-1 knockout (KO) mice as determined by RT-qPCR and immunohistochemistry (IHC) staining. Depigmented (KOD) and adjacent normal (KON) skin samples from Ets-1 KO mice (*n* = 6 per group) were subjected to RT-qPCR analysis. (**A**) A negative correlation was observed between Ct values obtained by RT-qPCR and the log_2_ FPKM values from RNA-seq analysis. (**B**) RT-qPCR quantification of melanogenesis-related genes, including tyrosinase (*Tyr*), tyrosinase-related protein 1 (*Tyrp1*), lymphoid enhancer binding factor 1 (*Lef1*), dopachrome tautomerase (*Dct*), frizzled class receptor 1 (*Fzd1*), endothelin receptor type B (*Ednrb*), sex-determining region Y-box 9 (*Sox9*), and microphthalmia-associated transcription factor (*Mift*). * and ** indicates *P* < 0.05 and *P* < 0.01, respectively. (**C**, **D**) IHC staining of Tyr protein in KON (**C**) and KOD (**D**) skin sections. Arrows and the zoomed-in inset in (**C**) highlight Tyr-positive signals primarily localized in the dermal papilla and matrix regions of hair follicles in normal skin. Tyr staining is absent in the corresponding region of depigmented skin. Bars, 100 μm

To validate the transcriptional changes observed in the depigmented skin of Ets-1 KO mice, RT-qPCR was performed to assess the expression of key melanogenesis-related genes. Total RNA was extracted from both depigmented and adjacent pigmented skin tissues of Ets-1 KO mice and analyzed via RT-qPCR. A strong negative correlation was observed between the Ct values obtained from RT-qPCR and the log_2_ FPKM values from RNA-seq analysis, confirming the consistency between the two methods (Fig. 3A). Compared to the pigmented skin, the depigmented areas of Ets-1 KO mice exhibited significantly downregulated expression of several melanogenesis-associated genes, including *Tyr*, *Tyrp1*, *Lef1*, *Dct*, *Fzd1*, *Ednrb*, *Sox9*, and *Mitf* (Fig. 3B). Although motif scanning analysis indicated potential Ets-1 binding sites in the promoter regions of mouse *Tyr* and *Dct* genes (q-values < 0.05; Supplementary Table S4), further functional studies are needed to confirm direct transcriptional regulation by Ets-1. Moreover, IHC staining supported the RT-qPCR findings, revealing a marked reduction in Tyr protein expression, a key enzyme in melanogenesis, in the depigmented skin of Ets-1 KO mice (Fig. 3D). In contrast, strong Tyr antigenicity was detected in the dermal papilla and matrix regions of hair follicles in adjacent pigmented areas (Fig. 3C). The absence of Tyr expression in the depigmented skin may reflect either a depletion of MCs or impaired Tyr expression within existing MCs, These results collectively suggest a disruption of melanogenesis and pigment production in the absence of Ets-1.

### PPI network analysis

**Fig. 4:**
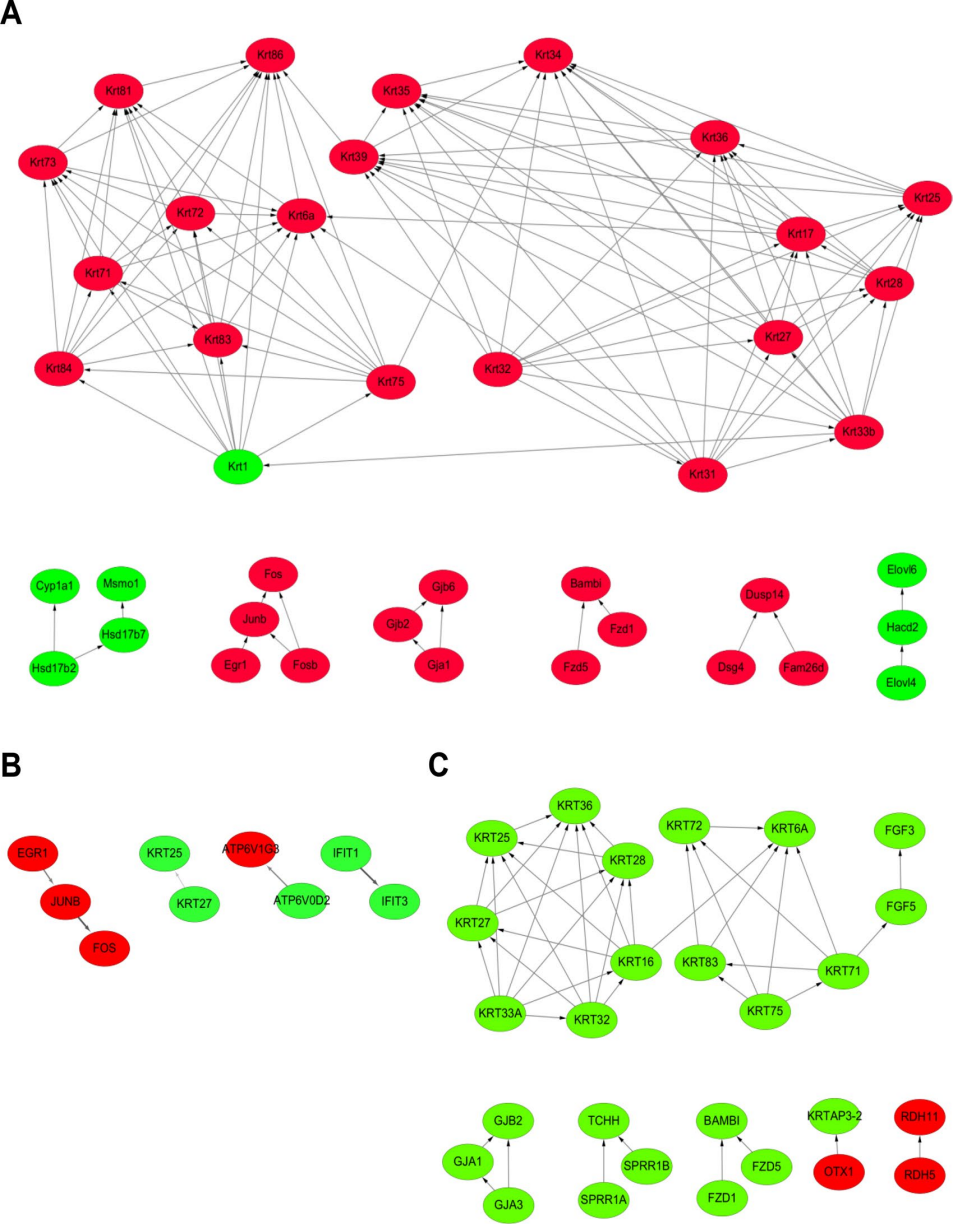
Protein-protein interaction (PPI) network analysis. PPI networks were constructed using Cytoscape, with all DEGs mapped to the STRING human protein database. (**A**) PPI network analysis of normal skin from Ets-1 knockout (KO) mice compared to wild-type (WT) counterparts. (**B**) PPI network analysis of depigmented skin of Ets-1 KO mice compared to WT mouse skin. (**C**) PPI network analysis of depigmented versus pigmented skins from Ets-1 KO mice. Ellipses represent proteins encoded by upregulated (red) and downregulated (green) genes

To further elucidate the interactions among the DEGs identified in the comparison of Ets-1 KO and WT mouse skins, we integrated gene expression profiles with whole-genome PPI data to construct PPI networks. These networks were built using the STRING human database and compared DEGs between three groups: (1) the normal skins of WT and KO mice, (2) the depigmented skin of KO mice and the normal skin of WT mice, and (3) the depigmented and normal skins of KO mice. The results of the PPI network analysis are illustrated in Fig. 4. The network constructed from the comparison between the normal skins of WT and KO mice identified key hub proteins, including KRT25, KRT27, EGR1, JUNB, FOS, ATP6V0D2, ATP6V1G3, IFIT1, and IFIT3 as central nodes (Fig. 4A), suggesting their potential roles in melanogenesis and skin pigmentation. Interestingly, the network comparison between the depigmented skin of KO mice and the normal skin of WT mice revealed a similar set of hub genes (Fig. 4B), indicating consistent molecular alterations in the normal skin of KO mice compared to WT controls. Notably, the comparison between the depigmented and the normal skins of KO mice identified a main network highlighting interactions among keratin proteins, extending to FGF5 and FGF3, as well as additional discrete interactions (Fig. 4C). This emphasizes the network differences within the KO mice. Collectively, these networks underscore the complex regulatory mechanisms underlying the depigmentation phenotype in Ets-1 KO mice, with EGR1, JUNB, and FOS emerging as critical regulators. The interconnected clusters in the PPI networks suggest coordinated regulation of melanogenesis, stress response, and cellular signaling pathways. This analysis provides valuable insights into potential therapeutic targets for conditions involving MC dysfunction, while further research is needed to fully elucidate the molecular basis of Ets-1 deficiency in skin pigmentation disorders.

## Discussion

A systems biology approach provides a comprehensive framework for understanding disease mechanisms and facilitates the identification of novel therapeutic targets as well as opportunities for drug repurposing. Although skin hypopigmentation has previously been reported in *Ets-1* null mice [20], this study is, to our knowledge, the first to characterize the vitiligo-like phenotype in these mice through detailed histological and transcriptomic analyses. The spontaneous depigmentation observed in the ventral trunk and distal limbs, along with the histological characteristics showing reduced melanosome granules, as well as the downregulation of melanogenesis-related genes—particularly *Tyr* at both the transcript and protein levels—strongly support the conclusion that *Ets-1* deficiency impairs melanogenesis. Previous studies have implicated *Ets-1* in cranial neural crest migration in chick embryos [21] and in cardiac neural crest migration and differentiation in Ets-1 KO mice [22]. In addition, Ets-1 has been shown to activate an enhancer essential for Sox10 expression in neural crest-derived lineages, including MCs [20]. Furthermore, the role of interstitial MMPs, which also regulated by Ets-1, in melanoblast migration and tissue remodeling is well established [23]. The absence of Ets-1 expression in the outer root sheath of hair follicles in KO mice suggests that Ets-1 gene depletion may impair the migration of melanoblasts from the neural crest during key stages of cutaneous morphogenesis, thereby preventing the establishment of mature MCs in these regions. Alternatively, Ets-1 deficiency may interfere with melanoblast maturation into functional MCs, compromising their survival and ultimately contributing to the pathogenesis of vitiligo.

In addition to its established role in regulating interstitial MMP activity [13], accumulating evidence indicates that Ets-1 is transcriptionally responsive to redox-sensitive signaling pathways [24] and, in turn, modulates intracellular glutathione levels in cancer cells [25]. These findings suggest that Ets-1 may contribute to oxidative stress regulation in various cellular contexts. Supporting this idea, our recent observation of co-localization between Ets-1 and heme oxygenase-1―a key antioxidant enzyme―in hepatocytes of exosome-rescued murine livers [26] indirectly points to a cytoprotective role of Ets-1, which may also be relevant for MC survival, particularly in depigmentation disorders. Furthermore, IHC data from our study reveal that Ets-1 is predominantly expressed in epidermal keratinocytes. This spatial expression pattern supports the existence of a paracrine regulatory mechanism, wherein Ets-1 expression in keratinocytes influences melanogenesis and/or the survival of adjacent MCs by modulating the secretion of MC-supporting cytokines, such as α-melanocyte stimulating hormone and endothelin-1 [27]. These cytokines are known to promote melanin synthesis and protect MCs from environmental stress, indicating a potentially important functional link between Ets-1 and skin pigmentation homeostasis. Although alterations in capillary density have not been widely reported in vitiligo-affected skin, insights from developmental and cancer biology have identified Ets-1 as a significant regulator of vasculogenesis and tumor angiogenesis [21, 28]. Therefore, future investigations into changes in MC apoptosis and capillary density in Ets-1 KO mice―as well as in human depigmented skin―may help clarify potential cytoprotective and vascular regulatory functions of Ets-1 in vitiligo pathogenesis. Nevertheless, the precise molecular pathways through which Ets-1 coordinates MC biology and vascular dynamics remain to be fully elucidatd. A deeper understanding of these mechanisms could inform the development of novel therapeutic strategies aimed at enhancing MS survival and promoting skin repigmentation in vitiligo and other pigmentary disorders.

In the context of vitiligo pathogenesis, systemic and epidermal levels of IL-17 have been found to be significantly elevated in vitiligo patients, with increased IL-17 signaling proposed as a biomarker for disease activity [16, 29–35]. Mechanistically, IL-17 enhances Th17 response not only in peripheral blood but also in vitiligo skin lesions, creating a stressful microenvironment that promotes autophagic cell death of MCs [36]. Given the critical role of Th17 cytokines in T-cell function and their involvement in the pathogenesis of autoimmune and allergic diseases [37], Ets-1 has been identified as a key inhibitory transcription factor for Th17 differentiation [17]. Consequently, genetic depletion of Ets-1 is very likely to induce the elevation of Th17 signaling that facilitates disease development. In this study, KEGG pathway enrichment analysis in this study revealed a significant impact of Ets-1 deficiency on the IL-17 signaling pathway. Comparison of the DEGs between the depigmented and paralesional normal skins of Ets-1 KO mice showed significant upregulation of C-C motif chemokine ligand 2 (*CCL2*) and *CCL7* genes (supplementary Figure S2), both of which are implicated in the pathogenesis of autoimmune connective tissue diseases [38]. Although elevated CCL2 expression in vitiligo fibroblasts has recently been linked to promoting type 2 cytokine secretion [39], the detailed mechanisms await further elucidation. Notably, the coincident dysregulation observed in both melanogenesis and IL-17 signaling pathways in the depigmented skin lesions of Ets-1 KO mice supports the applicability of this model for studying vitiligo pathomechanisms and developing therapeutics strategies. However, whether IL-17 signaling is elevated in the depigmented and/or normal skins of Ets-1 KO mice requires further investigation.

Furthermore, in the list of the DEGs comparing the normal skin of KO and WT mice, we observed significant downregulation of numerous *keratin* and *Krtap* genes in the depigmented skin of KO mice. Given the essential roles of keratin proteins in maintaining the structural integrity and barrier function of the skin, these findings suggest that the downregulation of *Krtap* genes in depigmented skin may impair the formation of the intermediate filament network of keratins, thereby weakening mechanical strength and resilience. Additionally, the reduction in *Krtap* gene expression may disrupt melanosome transfer from MCs to keratinocytes, contributing to hypopigmentation [40, 41]. Similarly, since keratins are involved in the proliferation, differentiation, and apoptosis of skin cells, the downregulation of *Krtap* genes in the skins or MCs may also play a role in delayed wound healing [42] and reduced responsiveness to UV radiation-induced oxidative stress [43] in vitiligo. However, the precise role of Krtap gene/protein in vitiligo pathogenesis warrants further investigation.

In the context of melanogenesis pathway, the Wnt/β-catenin signaling axis plays a critical role in regulating the transcription of the *Mitf* gene, which in turn controls the expression of melanogenesis-related enzymes, including Tyr, Tryp1, and Dct. The KEGG pathway enrichment analysis in this study revealed significant downregulation of several Wnt signaling mediator genes, including Wnt ligand, Frizzled receptor, and TCF/LEF transcription factor, in the depigmented skin of Ets-1 KO mice. Given that Wnt/β-catenin signaling pathway is downregulated in vitiligo and that its activation promotes MC regeneration by driving the differentiation of melanoblasts into MCs [44, 45], the repressed expression of Wnt signaling mediator genes in KO mice underscores the clinical relevance of this animal model in recapitulating the molecular features of vitiligo.

The present study, which utilized Ets-1 deficient mice as a model for studying vitiligo, has several limitations. First, the transcriptome data derived from RNA extracts of whole skin tissues may not accurately represent the transcriptomes of specific cell types, such as epithelial or dermal cells. Advanced single-cell transcriptomic analysis could help address this limitation. Second, the PPI networks proposed in this study were constructed under the assumption that mRNA and protein expression levels are positively correlated. However, the relationship between transcription and translation requires further validation. Third, mice and humans exhibit significant genetic and physiological differences. Although Ets-1 deficient mice display a vitiligo-like phenotype, the underlying mechanisms and pathways may not fully replicate those in human vitiligo, limiting the direct applicability of findings from mouse models to human patients. Additionally, while preclinical models provide powerful tools for studying disease pathogenesis and developing new treatments, the complexity of vitiligo pathogenesis poses challenges. Vitiligo is a multifactorial disease involving genetic, environmental, and immunological factors, and Ets-1 deficient mice may not fully capture this complexity, potentially oversimplifying the disease model. Therefore, these limitations should be carefully considered when interpreting the results and designing follow-up mechanistic and therapeutic studies.

## Conclusions

This study verifies that the vitiligo-like skin depigmentation in Ets-1 deficient mice may be attributed to Ets-1 gene depletion, which may play a pivotal role in the pathogenesis of vitiligo. We also propose that the therapeutic effect of topical Ets-1 gene delivery in the depigmented skins of KO mice or topical agonistic treatment could be further studied, which hopefully develop a novel vitiligo treatment tool by restoring its cutaneous expression and function.

## Electronic Supplementary Material

Below is the link to the electronic supplementary material.

Supplementary Material 1

Supplementary Material 2

## Data Availability

All data generated or analysed during this study are available from the corresponding author on reasonable request.

## Abbreviations

CCL: C-C motif chemokine ligand
Dct: Dopachrome tautomerase
DEGs: Differentially expressed genes
Ednrb: Endothelin receptor type B
ERK: Extracellular signal-regulated kinase
FDR: False discovery rate
FGF: Fibroblast growth factor
FPKM: Fragments per kilobase of transcript per million
Fzd1: Frizzled class receptor 1
H&E: Hematoxylin and eosin
IFIT: Interferon induced protein
IHC: Immunohistochemistry
IL-17: Interleukin-17
KEGG: Kyoto encyclopedia of genes and genomes
KO: Knockout
KRT: Keratin
Krtap: Keratin-associated protein
Lef1: Lymphoid enhancer binding factor 1
MCs: Melanocytes
Mitf: Microphthalmia-associated transcription factor
MMP: Matrix metalloproteinase
NGS: Next-generation sequencing
PPI: Protein-protein interaction
Sox9: SRY (sex determining region Y)-box 9
Tyr: Tyrosinase
Tyrp1: Tyrosinase-related protein 1
WT: Wild-type

## References

[1] Essien KI, Harris JE. Animal models of vitiligo: matching the model to the question. Dermatol Sinica. 2014;32:240–47.

[2] Koga M. Vitiligo: a new classification and therapy. Br J Dermatol. 1977;97:255–61. 921895 10.1111/j.1365-2133.1977.tb15180.x

[3] Koga M, Tango T. Clinical features and course of type A and type B vitiligo. Br J Dermatol. 1988;118:223–8. 3348967 10.1111/j.1365-2133.1988.tb01778.x

[4] el-Mofty AM, el-Mofty M. Vitiligo. A symptom complex. Int J Dermatol. 1980;19:237–44. 6993382 10.1111/j.1365-4362.1980.tb00316.x

[5] Sharrocks AD. The ETS-domain transcription factor family. Nat Rev Mol Cell Biol. 2001;2:827–37. 11715049 10.1038/35099076

[6] Pufall MA, Lee GM, Nelson ML, Kang HS, Velyvis A, Kay LE, et al. Variable control of Ets-1 DNA binding by multiple phosphates in an unstructured region. Science. 2005;309:142–5. 15994560 10.1126/science.1111915

[7] Bories JC, Willerford DM, Grevin D, Davidson L, Camus A, Martin P, et al. Increased T-cell apoptosis and terminal B-cell differentiation induced by inactivation of the Ets-1 proto-oncogene. Nature. 1995;377:635–8. 7566176 10.1038/377635a0

[8] Leng RX, Pan HF, Chen GM, Feng CC, Fan YG, Ye DQ, et al. The dual nature of Ets-1: focus to the pathogenesis of systemic lupus erythematosus. Autoimmun Rev. 2011;10:439–43. 21296190 10.1016/j.autrev.2011.01.007

[9] Behrens P, Rothe M, Wellmann A, Krischler J, Wernert N. The Ets-1 transcription factor is up-regulated together with MMP 1 and MMP 9 in the stroma of pre-invasive breast cancer. J Pathol. 2001;194:43–50. 11329140 10.1002/path.844

[10] Luchtel RA. ETS1 function in leukemia and lymphoma. Adv Exp Med Biol. 2024;1459:359–78. 39017852 10.1007/978-3-031-62731-6_16

[11] Nelson KK, Subbaram S, Connor KM, Dasgupta J, Ha XF, Meng TC, et al. Redox-dependent matrix metalloproteinase-1 expression is regulated by JNK through Ets and AP-1 promoter motifs. J Biol Chem. 2006;281:14100–10. 16569638 10.1074/jbc.M601820200

[12] Rothhammer T, Hahne JC, Florin A, Poser I, Soncin F, Wernert N, et al. The Ets-1 transcription factor is involved in the development and invasion of malignant melanoma. Cell Mol Life Sci. 2004;61:118–28. 14704859 10.1007/s00018-003-3337-8 PMC11138723

[13] Kumar R, Parsad D, Kanwar AJ, Kaul D. Altered levels of Ets-1 transcription factor and matrix metalloproteinases in melanocytes from patients with vitiligo. Br J Dermatol. 2011;165:285–91. 21428970 10.1111/j.1365-2133.2011.10324.x

[14] Srivastava N, Bishnoi A, Mehta S, Rani S, Kumar R, Bhardwaj S, et al. Aberrant ETS-1 signalling impedes the expression of cell adhesion molecules and matrix metalloproteinases in non-segmental vitiligo. Exp Dermatol. 2020;29:539–47. 32350934 10.1111/exd.14107

[15] Eby JM, Kang HK, Klarquist J, Chatterjee S, Mosenson JA, Nishimura MI, et al. Immune responses in a mouse model of vitiligo with spontaneous epidermal de- and repigmentation. Pigment Cell Melanoma Res. 2014;27:1075–85. 24935676 10.1111/pcmr.12284 PMC4470702

[16] Bassiouny DA, Shaker O. Role of interleukin-17 in the pathogenesis of vitiligo. Clin Exp Dermatol. 2011;36:292–7. 21198791 10.1111/j.1365-2230.2010.03972.x

[17] Moisan J, Grenningloh R, Bettelli E, Oukka M, Ho IC. Ets-1 is a negative regulator of Th17 differentiation. J Exp Med. 2007;204:2825–35. 17967903 10.1084/jem.20070994 PMC2118518

[18] Grenningloh R, Kang BY, Ho IC. Ets-1, a functional cofactor of T-bet, is essential for Th1 inflammatory responses. J Exp Med. 2005;201:615–26. 15728239 10.1084/jem.20041330 PMC2213045

[19] Kao YH, Chen CL, Jawan B, Chung YH, Sun CK, Kuo SM, et al. Upregulation of hepatoma-derived growth factor is involved in murine hepatic fibrogenesis. J Hepatol. 2010;52:96–105. 19913322 10.1016/j.jhep.2009.10.002

[20] Saldana-Caboverde A, Perera EM, Watkins-Chow DE, Hansen NF, Vemulapalli M, Mullikin JC, et al. The transcription factors Ets1 and Sox10 interact during murine melanocyte development. Dev Biol. 2015;407:300–12. 25912689 10.1016/j.ydbio.2015.04.012 PMC4618791

[21] Tahtakran SA, Selleck MA. Ets-1 expression is associated with cranial neural crest migration and vasculogenesis in the chick embryo. Gene Expr Patterns. 2003;3:455–8. 12915311 10.1016/s1567-133x(03)00065-6

[22] Gao Z, Kim GH, Mackinnon AC, Flagg AE, Bassett B, Earley JU, et al. Ets1 is required for proper migration and differentiation of the cardiac neural crest. Development. 2010;137:1543–51. 20356956 10.1242/dev.047696 PMC2853851

[23] Nagase H, Woessner JF. Jr. Matrix metalloproteinases. J Biol Chem. 1999;274:21491–4. 10419448 10.1074/jbc.274.31.21491

[24] Wilson LA, Gemin A, Espiritu R, Singh G. ets-1 is transcriptionally up-regulated by H2O2 via an antioxidant response element. FASEB J. 2005;19:2085–7. 16234432 10.1096/fj.05-4401fje

[25] Verschoor ML, Singh G. Ets-1 regulates intracellular glutathione levels: key target for resistant ovarian cancer. Mol Cancer. 2013;12:138. 24238102 10.1186/1476-4598-12-138 PMC3842663

[26] Kao YH, Chang CY, Lin YC, Chen PH, Lee PH, Chang HR, et al. Mesenchymal stem cell-derived exosomes mitigate acute murine liver injury via Ets-1 and Heme oxygenase-1 up-regulation. Curr Stem Cell Res Ther. 2024;19:906–18. 37723631 10.2174/1574888X19666230918102826

[27] Yuan XH, Jin ZH. Paracrine regulation of melanogenesis. Br J Dermatol. 2018;178:632–39. 28494100 10.1111/bjd.15651

[28] Wang S, Wan L, Zhang X, Fang H, Zhang M, Li F, et al. ETS-1 in tumor immunology: implications for novel anti-cancer strategies. Front Immunol. 2025;16:1526368. 40181983 10.3389/fimmu.2025.1526368 PMC11965117

[29] Bhardwaj S, Rani S, Srivastava N, Kumar R, Parsad D. Increased systemic and epidermal levels of IL-17A and IL-1beta promotes progression of non-segmental vitiligo. Cytokine. 2017;91:153–61. 28082234 10.1016/j.cyto.2016.12.014

[30] Elela MA, Hegazy RA, Fawzy MM, Rashed LA, Rasheed H. Interleukin 17, Interleukin 22 and FoxP3 expression in tissue and serum of non-segmental vitiligo: a case- controlled study on eighty-four patients. Eur J Dermatol. 2013;23:350–5. 23797460 10.1684/ejd.2013.2023

[31] Chatterjee S, Eby JM, Al-Khami AA, Soloshchenko M, Kang HK, Kaur N, et al. A quantitative increase in regulatory T cells controls development of vitiligo. J Invest Dermatol. 2014;134:1285–94. 24366614 10.1038/jid.2013.540 PMC3989443

[32] Speeckaert R, Speeckaert M, De Schepper S, van Geel N. Biomarkers of disease activity in vitiligo: A systematic review. Autoimmun Rev. 2017;16:937–45. 28698094 10.1016/j.autrev.2017.07.005

[33] Singh RK, Lee KM, Vujkovic-Cvijin I, Ucmak D, Farahnik B, Abrouk M, et al. The role of IL-17 in vitiligo: A review. Autoimmun Rev. 2016;15:397–404. 26804758 10.1016/j.autrev.2016.01.004 PMC4769658

[34] Zhen Y, Yao L, Zhong S, Song Y, Cui Y, Li S. Enhanced Th1 and Th17 responses in peripheral blood in active non-segmental vitiligo. Arch Dermatol Res. 2016;308:703–10. 27687555 10.1007/s00403-016-1690-3

[35] Wang CQ, Cruz-Inigo AE, Fuentes-Duculan J, Moussai D, Gulati N, Sullivan-Whalen M, et al. Th17 cells and activated dendritic cells are increased in vitiligo lesions. PLoS ONE. 2011;6:e18907. 21541348 10.1371/journal.pone.0018907 PMC3081835

[36] Zhou J, An X, Dong J, Wang Y, Zhong H, Duan L, et al. IL-17 induces cellular stress microenvironment of melanocytes to promote autophagic cell apoptosis in vitiligo. FASEB J. 2018:4899–916. 10.1096/fj.201701242RR 29613836

[37] Bettelli E, Oukka M, Kuchroo VK. T(H)-17 cells in the circle of immunity and autoimmunity. Nat Immunol. 2007;8:345–50. 17375096 10.1038/ni0407-345

[38] Bujor AM, El Adili F, Parvez A, Marden G, Trojanowska M. Fli1 downregulation in scleroderma myeloid cells has profibrotic and Proinflammatory effects. Front Immunol. 2020;11:800. 32508810 10.3389/fimmu.2020.00800 PMC7248379

[39] Jin R, Zhou M, Lin F, Xu W, Xu A. Pathogenic Th2 cytokine profile skewing by IFN-γ-Responding vitiligo fibroblasts via CCL2/CCL8. Cells. 2023;12:217. 36672151 10.3390/cells12020217 PMC9856695

[40] Planko L, Bohse K, Hohfeld J, Betz RC, Hanneken S, Eigelshoven S, et al. Identification of a keratin-associated protein with a putative role in vesicle transport. Eur J Cell Biol. 2007;86:827–39. 17397964 10.1016/j.ejcb.2007.02.004

[41] Yamaguchi Y, Brenner M, Hearing VJ. The regulation of skin pigmentation. J Biol Chem. 2007;282:27557–61. 17635904 10.1074/jbc.R700026200

[42] Gupta A, Chauhan A, Priya A, Mantri B, Wadhokar M, Dalave K, et al. Lesional skin in vitiligo exhibits delayed in vivo reepithelialization compared to the nonlesional skin. Wound Repair Regen. 2020;28:307–14. 32003499 10.1111/wrr.12798

[43] Saleh FY, Awad SS, Nasif GA, Halim C. Epithelial expression of cytokeratins 15 and 19 in vitiligo. J Cosmet Dermatol. 2016;15:312–17. 27139521 10.1111/jocd.12223

[44] Lin X, Meng X, Lin J. The possible role of Wnt/beta-catenin signalling in vitiligo treatment. J Eur Acad Dermatol Venereol. 2023;37:2208–21. 36912722 10.1111/jdv.19022

[45] Birlea SA, Costin GE, Roop DR, Norris DA. Trends in regenerative medicine: repigmentation in vitiligo through melanocyte stem cell mobilization. Med Res Rev. 2017;37:907–35. 28029168 10.1002/med.21426 PMC5466503

