# Supplementary material for: Characterization of Ets-1 deficiency-induced depigmentation in a mouse model: insights into vitiligo pathogenesis
Source: Lab Anim Res. 2025 Nov 28;41:29. doi: 10.1186/s42826-025-00260-8 (PMC12661662; doi:10.1186/s42826-025-00260-8)
Supplement: Supplementary file 1 — Supplementary Material 1 [file 42826_2025_260_MOESM1_ESM.pdf]

Supplemental Figures

Figure S1.

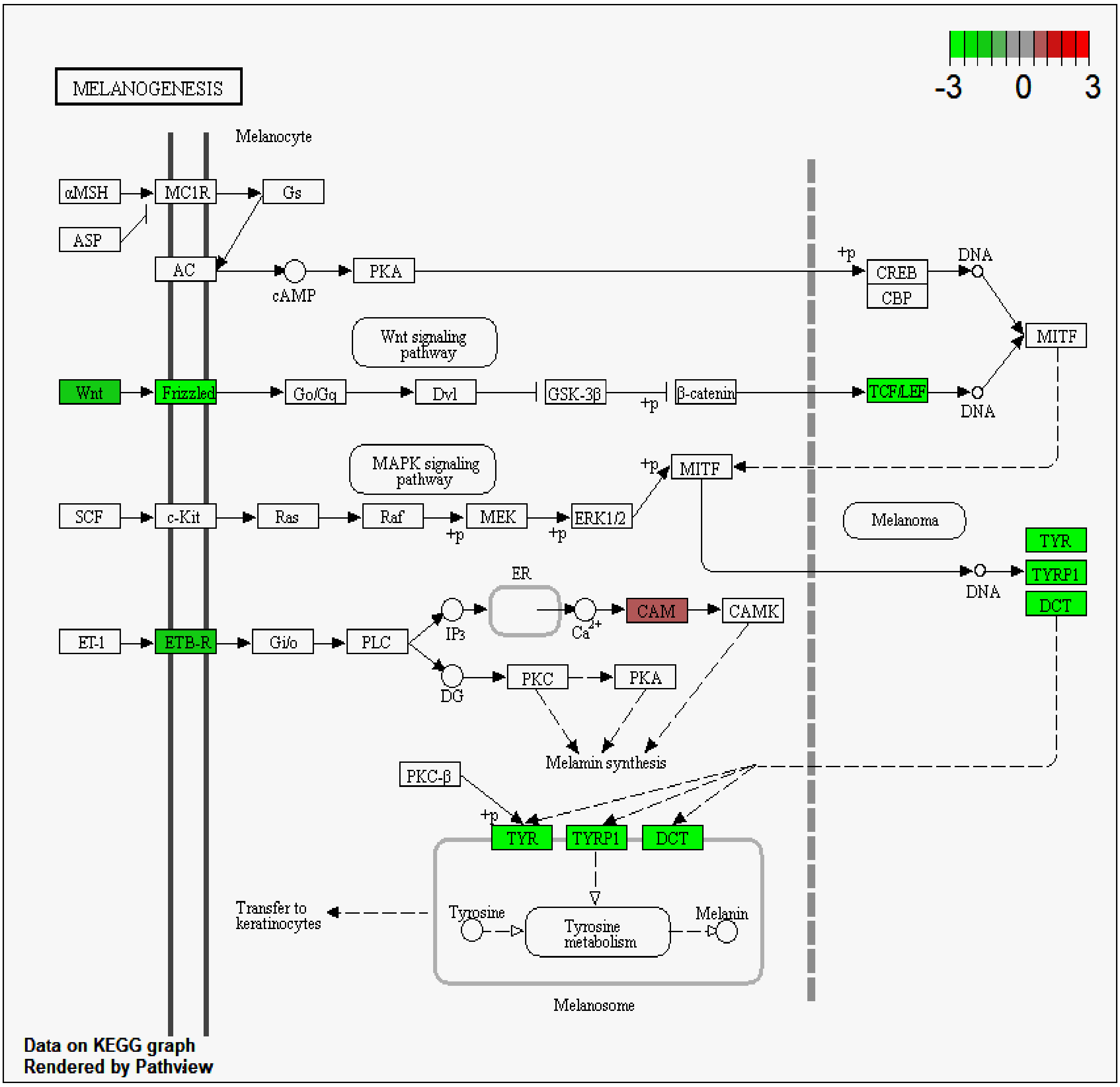

Figure S1. KEGG pathway enrichment analysis of DEGs in the depigmented skin of Ets-1 KO mouse compared to its normal skin. The results in melanogenesis pathway showed that the upregulated gene is calmodulin (CAM) shown in red box, while the downregulated genes shown in green boxes include Wnt, Frizzled class receptor 1 (Frizzled), T-cell factor/lymphoid enhancer factor (TCF/LEF), endothelin-1 receptor (ETB-R), tyrosinase (TYR), tyrosinase-related protein 1 (TYRP1), dopachrome tautomerase (DCT).

Figure S2.

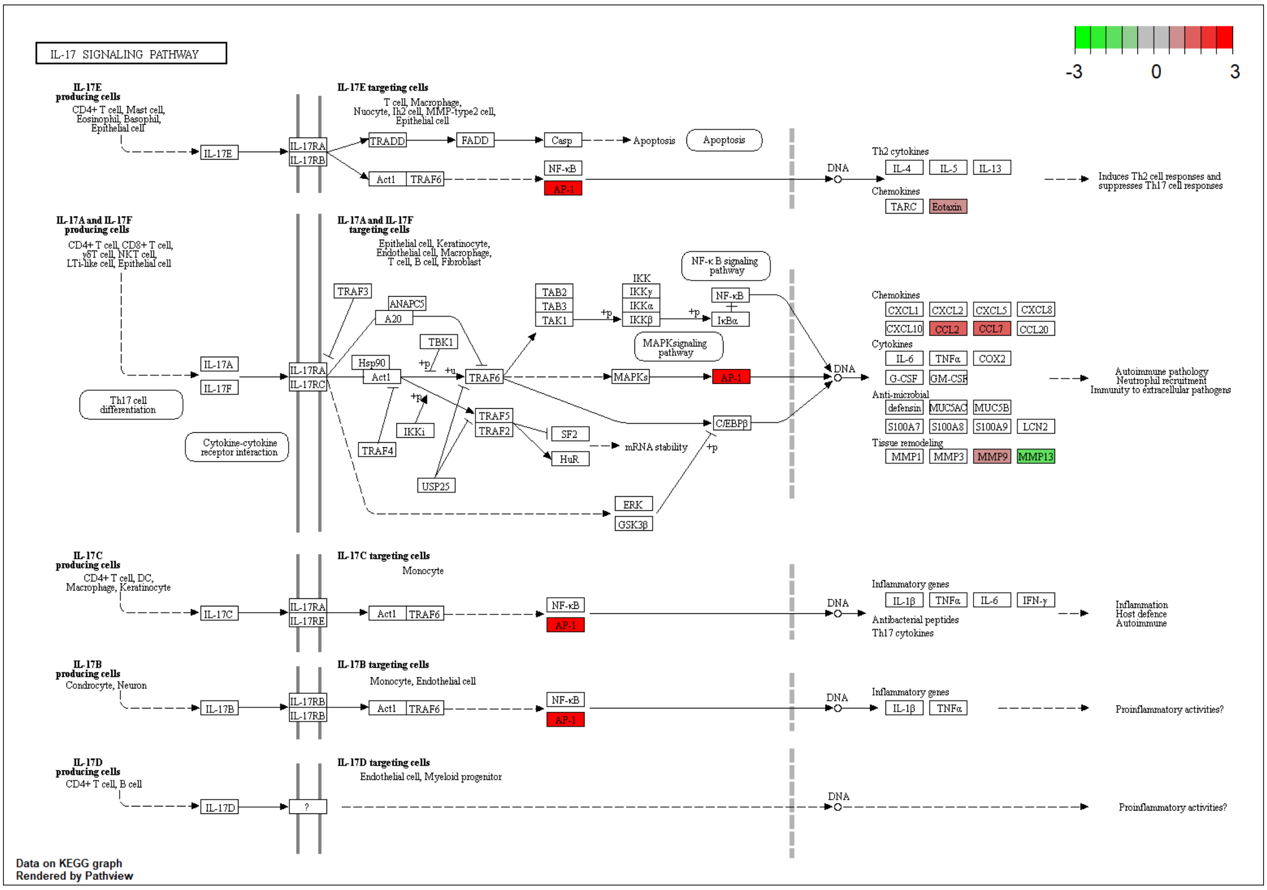

Figure S2. KEGG pathway enrichment analysis of DEGs in the depigmented skin of Ets-1 KO mouse compared to its normal skin. The results in IL-17 signaling pathway showed that the upregulated genes are activator protein 1 (AP-1), Eotarin, C-C motif chemokine ligand 2 (CCL2), CCL7, matrix metalloproteinase 9 (MMP9) shown in red boxes, while only MMP13 gene is downregulated shown in green box.
