# Supplementary material for: Characterization of Ets-1 deficiency-induced depigmentation in a mouse model: insights into vitiligo pathogenesis
Source: Lab Anim Res. 2025 Nov 28;41:29. doi: 10.1186/s42826-025-00260-8 (PMC12661662; doi:10.1186/s42826-025-00260-8)
Supplement: Supplementary file 2 — Supplementary Material 2 [file 42826_2025_260_MOESM2_ESM.pdf]

Supplementary Tables

Table S1 Characteristics of PCR primers and conditions

Genes	Sense primer (5' → 3')	Anti-sense primer (5' → 3')	Annealing temperature (°C)	Amplicon size (bp)
Tyr	CTCTATAGAAATGGTGATTTC	TCCATGAGGAGTGGCTG	56	266
Tyrp1	TATGACCCTGCTGTTCGAAG	CCAGAATGGCACCATGTTGT	60	228
Lef1	GAATGACAGCTGCCTACATC	TAGCAGTGACCTCAGGGTAA	56	187
Dct	GCAAGATTGCCTGTCTCTCCAG	CTTGAGAGTCCAGTGTTCCGTC	60	119
Fzd1	TGGCCCAGAGCTGCAAGAG	TCGTGTAGAACTTCCTCCAG	64	198
Ednrb	GCTCTGTATTTGGTGAGCAA	CGGAAGTTGTCATATCCGTG	56	149
Sox9	CACACGTCAAGCGACCCATGAA	TCTTCTCGCTCTCGTTCAGCAG	60	147
Mitf	TGGAAGACATCCTGATGGAC	CTCCTCCGGCTGCTTGTTTT	56	103
β-actin	ACCACAGCTGAGAGGGAAATCG	AGAGGTCTTTACGGATGTCAACG	60	277

Abbreviations: *Tyr*: tyrosinase; *Tyrp1*: tyrosinase-related protein 1; *Lef1*: lymphoid enhancer binding factor 1; *Dct*: dopachrome tautomerase; *Fzd1*: frizzled class receptor 1; *Ednrb*: endothelin receptor type B; *Sox9*: SRY-box transcription factor 9; *Mitf*: melanocyte inducing transcription factor

Table S2. KEGG pathway enrichment analysis of the differentially expressed genes (DEGs) in the pigmented skin of Ets-1 knockout mice as compared with wild-type mice.

Pathway	DEG no. (% of total 311 DEGs)	Genes in pathway (% total 8275 annotated genes)	P-value	Q-value *	Pathway ID
Estrogen signaling pathway	20 (6.43%)	133 (1.61%)	0.0000000996	0.0000236890	mmu04915
Biosynthesis of unsaturated fatty acid	8 (2.57%)	28 (0.34%)	0.0000000584	0.0005259845	mmu01040
Retinol metabolism	14 (4.50%)	91 (1.10%)	0.0000070305	0.0005270514	mmu00830
Fatty acid elongation	8 (2.57%)	32 (0.39%)	0.0000668056	0.0010256148	mmu00062
Steroid biosynthesis	6 (1.93%)	19 (0.23%)	0.0000822681	0.0023444249	mmu00100
Hippo signaling pathway	16 (5.14%)	154 (1.86%)	0.0002658451	0.0083625489	mmu04390
Ovarian steroidogenesis	9 (2.89%)	57 (0.69%)	0.0002436423	0. 0083118124	mmu04913
Basal cell carcinoma	9 (2.89%)	63 (0.76%)	0.0005254876	0.0157658245	mmu05217
Graft-versus-host disease	9 (2.89%)	65 (0.78%)	0.0007584585	0.0176212325	mmu05332
Cytokine-cytokine receptor interaction	23 (7.39%)	296 (3.58%)	0.0076854984	0.0176658545	mmu04060
Wnt signaling pathway	14 (4.52%)	148 (1.79%)	0.0013923951	0.0283576998	mmu04310
Steroid hormone biosynthesis	10 (3.22%)	88 (1.06%)	0.0016524251	0.0323165845	mmu00140
Arachidonic acid metabolism	10 (3.22%)	89 (1.08%)	0.0018354254	0.0325118124	mmu00590
Sphingolipid metabolism	7 (2.25%)	48 (0.58%)	0.0019381589	0.0329944909	mmu00600
TNF signaling pathway	11 (3.54%)	110 (1.33%)	0.0027525621	0.0434215061	mmu04668
Fatty acid metabolism	7 (2.25%)	52 (0.63%)	0.0031254820	0.0460576998	mmu01212
Tyrosine metabolism	6 (1.93%)	40 (0.48%)	0.0035226818	0.0490576998	mmu00350
Melanogenesis	10 (3.22%)	100 (1.21%)	0.0042165845	0.0557165845	mmu04916

* Adjusted P-value depicting significant enrichment (Q-value<0.05) of the gene sets in the pathways.

Table S3. KEGG pathway enrichment analysis of the differentially expressed genes (DEGs) in the depigmented skins of Ets-1 knockout mice as compared with wild-type mouse.

Pathway	DEG no. (% of total 155 DEGs)	Genes in pathway (% total 8275 annotated genes)	P-value	Q-value *	Pathway ID
Graft-versus-host disease	11 (7.09%)	65 (0.78%)	0.0000000156	0.0000176256	mmu05332
Phagosome	15 (9.67%)	181 (2.19%)	0.0000000562	0.0001026148	mmu04145
Estrogen signaling pathway	12 (7.44%)	133 (1.61%)	0.0000001252	0.0004267825	mmu04915
Allograft rejection	8 (5.16%)	64 (0.77%)	0.0000058425	0.0009259845	mmu05330
Autoimmune thyroid disease	8 (5.16%)	79 (0.95%)	0.0001226584	0.0035215854	mmu05320
Human T-cell leukemia virus 1 infection	15 (9.67%)	282 (3.41%)	0.0002702305	0.0064270514	mmu05166
Antigen processing and presentation	8 (5.16%)	91 (1.09%)	0.0003226581	0.0064444249	mmu04612
Type I diabetes mellitus	7 (4.52%)	70 (0.85%)	0.0003658451	0.0064625489	mmu04940
Herpes simplex infection	12 (7.74%)	215 (2.59%)	0.0007236423	0. 0124118124	mmu05168
Viral myocarditis	7 (4.52%)	88 (1.06%)	0.0013254876	0.0204658245	mmu05416
Leishmaniasis	6 (3.87%)	67 (0.81%)	0.0015236581	0.0219524585	mmu05140
Natural killer cell mediated cytotoxicity	8 (5.16%)	118 (1.42%)	0.0016524251	0.0219625845	mmu04650
Osteoclast differentiation	8 (5.16%)	128 (1.55%)	0.0027123951	0.0338576998	mmu04380
Cytokine-cytokine receptor interaction	13 (8.38%)	296 (3.58%)	0.0036854984	0.0419658545	mmu04060
Human papillomavirus infection	15 (9.67%)	370 (4.47%)	0.0039354254	0.0419818124	mmu05165
TNF signaling pathway	7 (4.52%)	110 (1.33%)	0.0045525621	0.0455215061	mmu04668
IL-17 signaling pathway	6 (3.87%)	91 (1.09%)	0.0071381589	0.0674944909	mmu04657

* Adjusted P-value depicting significant enrichment (Q-value<0.05) of the gene sets in the pathways.

Table S4. Motif scanning analysis of Ets-1 binding sites in promoter regions of melanogenesis-related genes

Gene Symbol	Ensembl ID	Strand	Start	End	p-value	q-value	Matched Sequence
Tyr	ENSMUSG00000004651	-	+43	+57	1.17e-05	0.0232	CTCACTTCCTCTGAT
Tyr	ENSMUSG00000004651	+	-394	-380	4.96e-05	0.0527	TCCTTTTCCTCTCTC
Tyr	ENSMUSG00000004651	+	-203	-189	8.65e-05	0.0613	ACTTCTTCCTGTAA
Dct	ENSMUSG00000022129	+	-700	-686	8.2e-06	0.0174	ACCACATCCTGTATT
Ednrb	ENSMUSG00000022122	+	-446	-432	5.7e-05	0.0915	ACCTTTTCCTCTTCC
Ednrb	ENSMUSG00000022122	+	-914	-900	8.3e-05	0.0915	TCCACATCCTCCAAC

Motif scanning was conducted on the promoter regions of target genes, defined as −1000 bp to +100 bp relative to the transcription start site (TSS), to identify potential Ets-1 binding sites. The position weight matrix (PWM) for the Mus musculus Ets-1 motif (Matrix ID: MA0098.2) was retrieved from the JASPAR database (10th release, 2024). Promoter sequences were analyzed using the FIMO tool from the MEME Suite (version 5.5.7; http://meme-suite.org/meme/), with a significance threshold set at $p < 0.0001$. Motif hits with a corresponding q-value < 0.05 were considered statistically significant. No significant Ets-1 motif occurrences ($p < 0.0001$) were detected in the promoter regions of *Tyrp1*, *Lef1*, *Fzd1*, *Sox9*, and *Mitf genes*.
